# GI-19007, a Novel Saccharomyces cerevisiae-Based Therapeutic Vaccine against Tuberculosis

**DOI:** 10.1128/CVI.00245-17

**Published:** 2017-12-05

**Authors:** Thomas H. King, Crystal A. Shanley, Zhimin Guo, Donald Bellgrau, Timothy Rodell, Synthia Furney, Marcela Henao-Tamayo, Ian M. Orme

**Affiliations:** a GlobeImmune Inc., Louisville, Colorado, USA; b Mycobacteria Research Laboratories, Department of Microbiology, Immunology and Pathology, Colorado State University, Fort Collins, Colorado, USA; University of Florida

**Keywords:** BCG vaccine, recombinant yeast vaccine, T cells, Th1 cells, Th17 cells, therapeutic, therapeutic vaccine, tuberculosis

## Abstract

As yet, very few vaccine candidates with activity in animals against Mycobacterium tuberculosis infection have been tested as therapeutic postexposure vaccines. We recently described two pools of mycobacterial proteins with this activity, and here we describe further studies in which four of these proteins (Rv1738, Rv2032, Rv3130, and Rv3841) were generated as a fusion polypeptide and then delivered in a novel yeast-based platform (Tarmogen) which itself has immunostimulatory properties, including activation of Toll-like receptors. This platform can deliver antigens into both the class I and class II antigen presentation pathways and stimulate strong Th1 and Th17 responses. In mice this fusion vaccine, designated GI-19007, was immunogenic and elicited strong gamma interferon (IFN-γ) and interleukin-17 (IL-17) responses; despite this, they displayed minimal prophylactic activity in mice that were subsequently infected with a virulent clinical strain. In contrast, in a therapeutic model in the guinea pig, GI-19007 significantly reduced the lung bacterial load and reduced lung pathology, particularly in terms of secondary lesion development, while significantly improving survival in one-third of these animals. In further studies in which guinea pigs were vaccinated with BCG before challenge, therapeutic vaccination with GI-19007 initially improved survival versus that of animals given BCG alone, although this protective effect was gradually lost at around 400 days after challenge. Given its apparent ability to substantially limit bacterial dissemination within and from the lungs, GI-19007 potentially can be used to limit lung damage as well as facilitating chemotherapeutic regimens in infected individuals.

## INTRODUCTION

Infections caused by the facultative intracellular bacterium Mycobacterium tuberculosis now have become the number one cause of death due to an infectious disease and have surpassed mortality caused by HIV (1–3). In addition, increasing numbers of new cases are drug resistant (4). Unsurprisingly, most of the effort to develop new vaccines that will be superior to or at least boost the current BCG vaccine have focused on vaccines that can be administered prophylactically (5). The objective of prophylactic vaccines is to generate a state of acquired immunologic memory immunity that can provide an accelerated protective immune response upon exposure in the lungs to M. tuberculosis (6). In contrast, much less attention has been spent on developing therapeutic vaccines that could be used in individuals already exposed, and accordingly there are very few candidates for which this activity can be demonstrated.

As previously reported (7), we identified seven proteins that are recognized by T cells harvested from the lungs of chronically infected mice, three of which are involved in iron acquisition by the bacillus and four of which are involved in the well-defined response to stress and hypoxia, all driven by the environment within the degenerating lung granuloma. When delivered in a synthetic highly effective Th1-directed glycopyranosyl lipid adjuvant (GLA), both pools of proteins failed to limit the course of highly virulent Beijing clinical isolates of M. tuberculosis in aerosol-exposed guinea pigs, but both significantly reduced lung damage and granulomatous inflammation and, in particular, almost completely prevented the formation of secondary lesions. Although these structures do not become necrotic (the emerging cellular immunity prevents the influx of neutrophils, which are the base cause of this necrosis), they can become very large, contributing to the eventual fatal consolidation of the lung tissue (8).

It has long been accepted that a strong Th1 response to a vaccine candidate is necessary for efficacy, and there is also an emerging viewpoint that Th17 responses are an important component as part of the overall control of the cellular influx into sites of infection (9–12). This knowledge has driven the development of innovative new classes of vaccine adjuvants needed to generate such responses and includes the addition of components that can trigger innate systems, such as the Toll-like receptors (TLRs), that direct Th1 responses (13–17). These include synthetic adjuvants based on GLA, which have been shown to be effective in models of tuberculosis (TB) infection both prophylactically and therapeutically (18, 19).

In the current work, we describe studies in which we used a novel, yeast-based platform, Tarmogen, which activates dendritic cells and macrophage receptors, including TLR-2, TLR-4, TLR-6, CD14, Dectin-1, Dectin-2, DEC-205, and the mannose receptor. Tarmogen yeast cells strongly drive Th1 and Th17 responses and were previously shown to be highly effective in viral infection and tumor challenge models (20–23). Our previously described four hypoxia-driven proteins were expressed as a single polypeptide within Saccharomyces cerevisiae yeast to create a novel vaccine, designated GI-19007, which then was used to vaccinate mice and guinea pigs exposed to a relevant highly virulent Beijing strain of M. tuberculosis.

The results of this study showed that GI-19007 was immunogenic in mice and induced antigen-specific Th1 and Th17 responses, including a strong CD8^+^ interleukin-17-positive (IL-17^+^) component, but was ineffective as a prophylactic vaccine. However, in our guinea pig therapeutic vaccination model, three inoculations with GI-19007 reduced the bacterial load in the lungs nearly 10-fold by day 70 postexposure and significantly dampened the extent of lung damage and pathology. As also seen in studies using GLA as the adjuvant platform (7), this resulted in approximately one-third of these animals surviving for significantly longer periods. Improved survival was also seen in guinea pigs vaccinated with BCG prior to therapeutic vaccination with GI-19007, although this effect was slowly lost after ∼400 days of the study for unknown reasons.

## RESULTS

### Demonstration that the Tarmogen platform can vaccinate against tuberculosis in mice.

The Tarmogen yeast-based platform has been used extensively in vaccines against viruses (22) but not for bacterial vaccines. As an initial proof-of-principle study, we expressed the immunodominant antigen Ag85A in the platform and used this to vaccinate mice. After challenge with the laboratory strain H37Rv, we determined vaccine efficacy over the next 30 days compared to that of the BCG vaccine (see Fig. S1 in the supplemental material). The yeast-based vaccine was protective at both day 15 and day 30 (*P* < 0.03) but not to the extent seen in BCG-vaccinated controls, in which strong protection (*P* < 0.001) was seen by day 15 (Fig. S1A). Both the Tarmogen and BCG vaccines gave very strong and accelerated CD4 T cell gamma interferon (IFN-γ) responses (Fig. S1B). There was an accelerated response in the Ag85A group in terms of IL-17^+^ CD4 cells, with evidence for a secondary wave of Th17 responses on day 60 of the challenge infection (Fig. S1C).

### Immune response to GI-19007 in mice.

**FIG 1:**
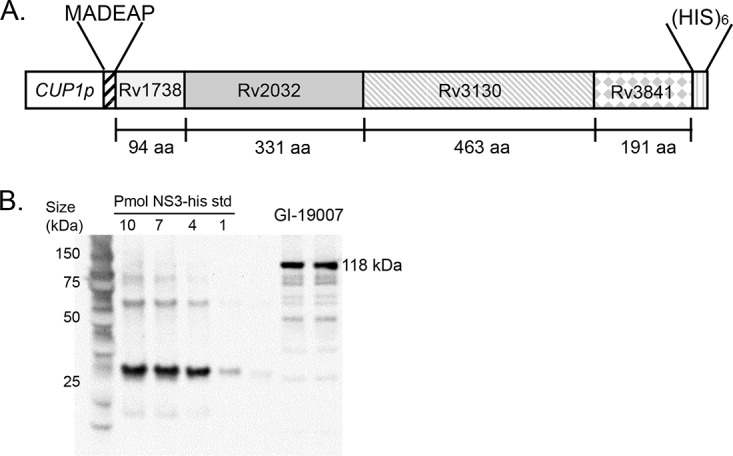
Structure and expression of the four target antigens in the yeast-based Tarmogen platform to construct the fusion protein vaccine GI-19007. (A) The genes encoding hypoxia antigens Rv1738, Rv2320, Rv3130, and Rv3841 were fused in frame and subcloned into a yeast 2μm expression plasmid under the control of the copper-inducible CUP1 promoter. A six-amino-acid (aa) N-terminal leader (MADEAP) was added for improved metabolic stability, and a C-terminal hexahistidine tag was included for antigen detection and quantification. (B) GI-19007 was cultured in medium lacking uridine (U2) and treated with 500 μM CuSO_4_ to induce protein antigen expression. Two micrograms of yeast lysate protein (GI-19007) and a dilution series of 1 to 10 pmol of His-tagged HCV protein standard (NS3-his std; observed molecular weight, 32) were subjected to SDS-PAGE and Western blot analysis with an anti-(His)_6_ tag monoclonal antibody; 1 YU = 10^7^ yeast cells. (Far left lane) Precision Plus protein standard with sizes listed in kDa. Expression levels of the ~118 kDa 4-hypoxia antigen ranged from 9,000 ng/YU to ~14,200 ng/YU (shown) for different lots of GI-19007.

**FIG 2:**
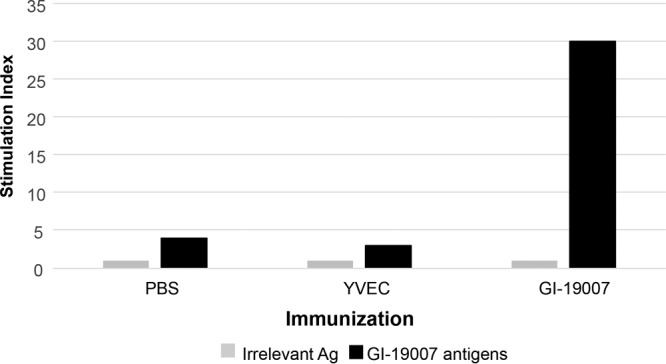
Immunization with GI-19007 elicits an antigen-specific IFN-γ response in mice. C57BL/6 mice were immunized intradermally with 2.5 YU per flank of GI-19007 or empty yeast vector (YVEC) on days 0 and 56. Ten days later, spleens were removed and stimulated in a 24-h ELISpot assay with 6 μg/ml of pooled recombinant antigens or the same concentration of irrelevant antigen. The ratio of IFN-γ ELISpot counts between the two are expressed as a stimulation index.

GI-19007 is a vaccine expressing a fusion of 4 hypoxia-driven TB antigens: Rv1738, Rv2032, Rv3130, and Rv3841. The ∼118-kDa fusion protein is expressed from the copper-inducible CUP1 promoter in Saccharomyces cerevisiae (Fig. 1). These antigens are thought to be part of an adaptation mechanism that enables bacterial persistence during developing necrosis. To determine the capacity of the GI-19007 vaccine to induce an IFN-γ response in mice, animals were vaccinated intradermally in the flanks twice, 56 days apart. Ten days later, responses to the fusion protein or to an irrelevant antigen (gelatin monomer) were determined in an enzyme-linked immunosorbent spot (ELISpot) assay (Fig. 2), demonstrating a strong IFN-γ response to the vaccine candidate. Additional studies showed that the response was indeed antigen specific, including identification of epitopes recognized by the host response (Fig. S2).

**FIG 3:**
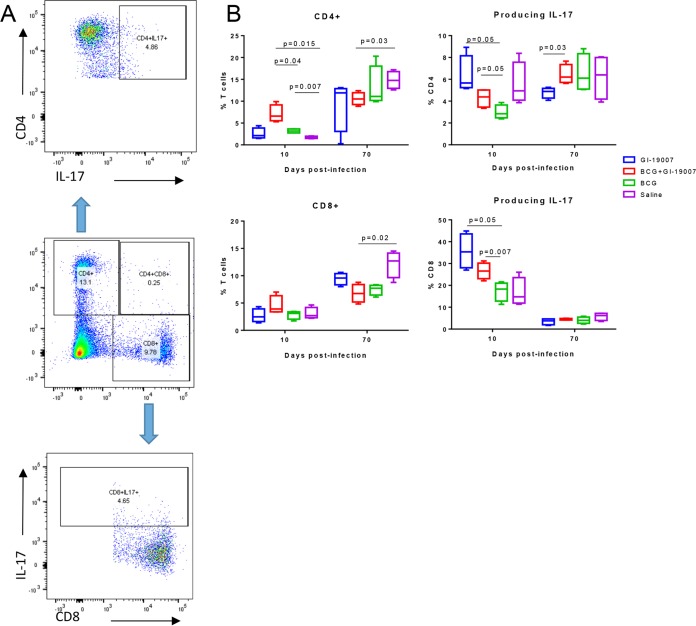
Vaccination of mice elicits a substantial IL-17 response. Mice were vaccinated with GI-19007 as described above and then challenged by low-dose aerosol with Beijing strain 212. Results are compared to those of controls given BCG or combination vaccination in a prime-boost format. Lung cells were harvested by tissue digestion and analyzed by flow cytometry. (A) Gating strategy. (B) GI-19007 induced strong Th17 responses, including within the CD8 T cell population.

Given previous data indicating the capacity of the yeast platform to strongly promote Th17 responses, we examined these responses in vaccinated mice early, or late, after exposure to low-dose aerosol infection with M. tuberculosis Beijing strain 212, using flow cytometry to analyze the host immune response to the yeast vector antigens and/or the hypoxia TB antigens expressed inside the Tarmogen system. The results of these flow-cytometric experiments showed a relatively even expansion of CD4 and CD8 cells in each of the vaccinated groups over 70 days of the infection (Fig. 3). As also noted above in the Ag85 vaccine study, we observed higher numbers of IL-17^+^ CD4 cells than we did with BCG-vaccinated mice (*P* < 0.05) early during the infection, and this was particularly evident in the case of CD8^+^ cells, in which ∼35% of lung CD8 cells were positive for this cytokine. In both cases, no differences were seen in the total numbers of activated CD44^hi^ CD62L^lo^ T cells entering the lungs (data not shown). Collectively, these data indicate a significant IL-17 component in the host response to GI-19007.

Despite these strong indications of immunogenicity, in two separate studies in which mice were vaccinated with GI-19007 three times and challenged with two relevant Beijing strains 6 weeks later, we were unable to demonstrate a statistically significant reduction in the lung bacterial load (data not shown). This suggests that the four-component antigens in our fusion construct are not recognized by the initial expansion of protective T cells after aerosol exposure, presumably because during this early stage these proteins are as yet not being produced by the infecting bacilli.

### Therapeutic activity of GI-19007 in the guinea pig model.

**FIG 4:**
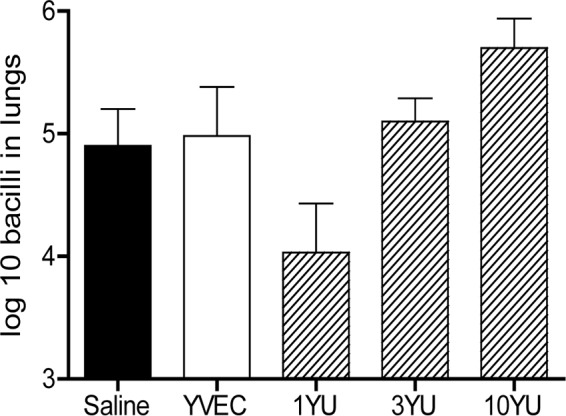
Dose ranging studies in guinea pigs. Animals were infected with approximately 10 Beijing strain 212 bacteria and then vaccinated with GI-19007 10, 25, and 40 days later. Empty platform (YVEC) was used as a negative control. The bacterial load in the lungs was determined on day 70. While the bacterial load in the lungs was significantly reduced (*P* < 0.03) by 0.9 log in animals given 1 YU of vaccine, no protection was seen at higher doses. Data shown as mean ± SEM (*n* = 5 guinea pigs).

**FIG 5:**
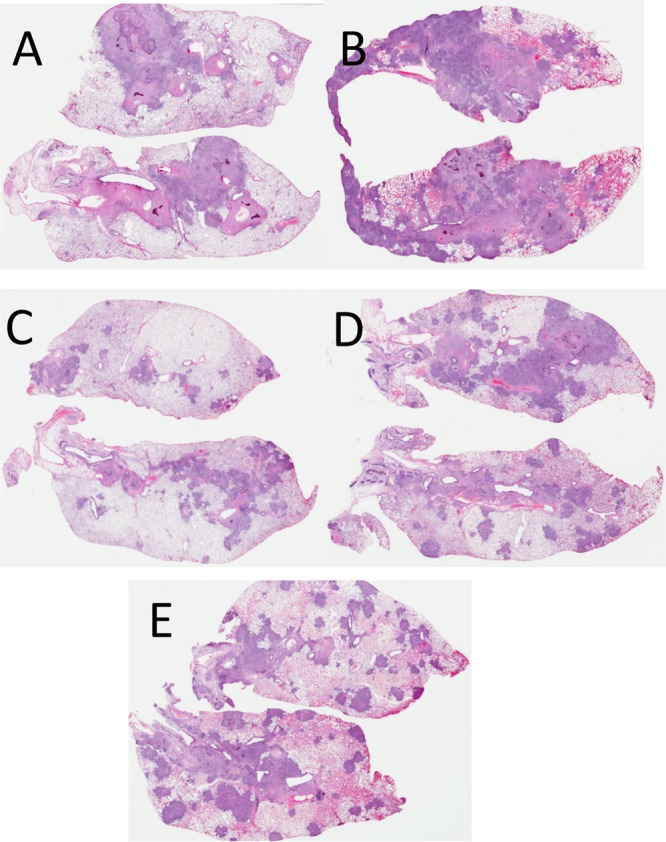
Whole-lung scans of representative samples stained with hematoxylin and eosin. (A) Saline control; (B) 3 YU YVEC control; (C) 1 YU GI-19007; (D) 3 YU GI-19007; (E) 10 YU GI-19007. Scans shown are from animals infected 70 days earlier with 10 to 20 M. tuberculosis Beijing strain 212 bacteria.

Given our earlier observations using the four antigens in a GLA-based adjuvant formulation, we decided to test GI-19007 in similar studies. Guinea pigs were infected with approximately 10 Beijing 212 bacteria and then vaccinated with GI-19007 10, 25, and 40 days later. On day 70, the bacterial load in the lungs was significantly reduced (*P* < 0.03) by 0.9 log in animals given 1 yeast unit (YU) of vaccine. However, in contrast, if the dose was increased to 3 YU this protection was lost, and when 10 YU was given the bacterial load was increased compared to that of saline controls (Fig. 4). When the histology of the lungs of these animals was evaluated, we noted that animals given 1 YU had far smaller lesions than controls and very much less secondary lesion development. This, however, was not the case when 3 or 10 YU was given, where lung damage was substantially worse (Fig. 5). In the lesions in animals receiving these higher doses there were substantial neutrophils present with significant degrees of lung damage and necrosis.

**FIG 6:**
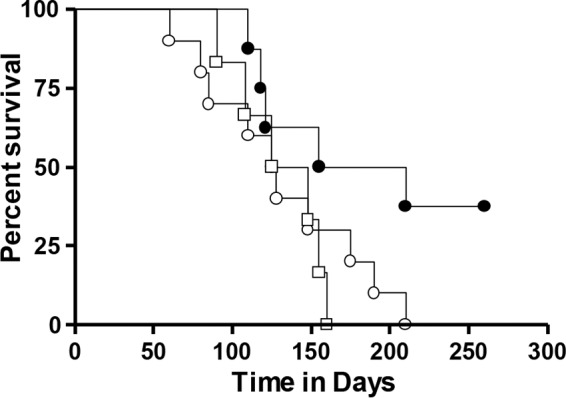
Kaplan-Meier survival curves for saline controls (open circles) and YVEC controls (open squares) versus animals vaccinated therapeutically with GI-19007 (closed circles). Because of curve overlap over the first 150 days, the curves were not significantly different, but substantially increased survival was seen in about one-third of the vaccinated animals past day 260. Each group contained 10 guinea pigs.

When survival of these groups of animals was evaluated, those given GI-19007 had patterns similar to those of saline and yeast empty vector control (YVEC) groups over the first ∼125 days, but while animals in the two control groups continued to trigger our Karnovsky scale, about 30% of the GI-19007 continued to survive past day 250 (Fig. 6). This result is very similar to our earlier observations using these four antigens.

### GI-19007 does not boost BCG but improves survival.

**FIG 7:**
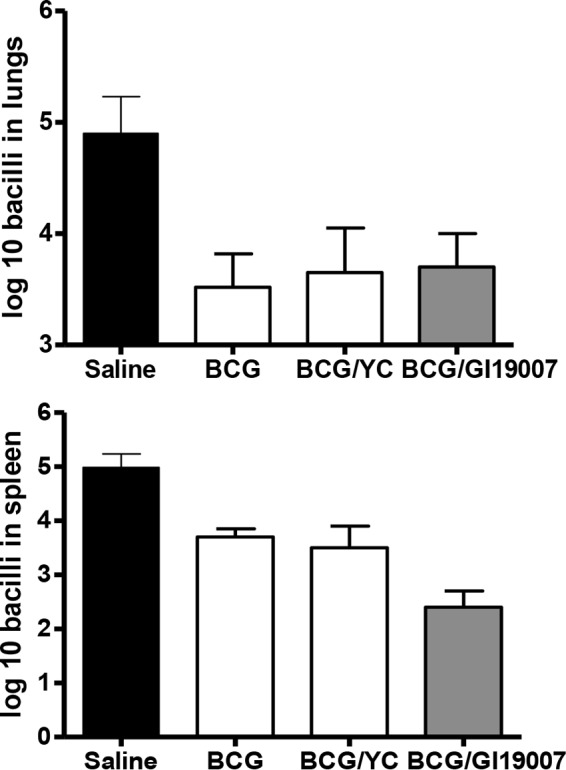
Demonstration that therapeutic vaccination with GI-19007 in guinea pigs that were previously given BCG vaccination prior to challenge with Beijing strain 212 did not improve protection in the lungs compared to BCG alone but significantly prevented dissemination to the spleen (*P* < 0.01). Data are shown as means ± standard errors of the means (SEM) (*n* = 5 guinea pigs).

**FIG 8:**
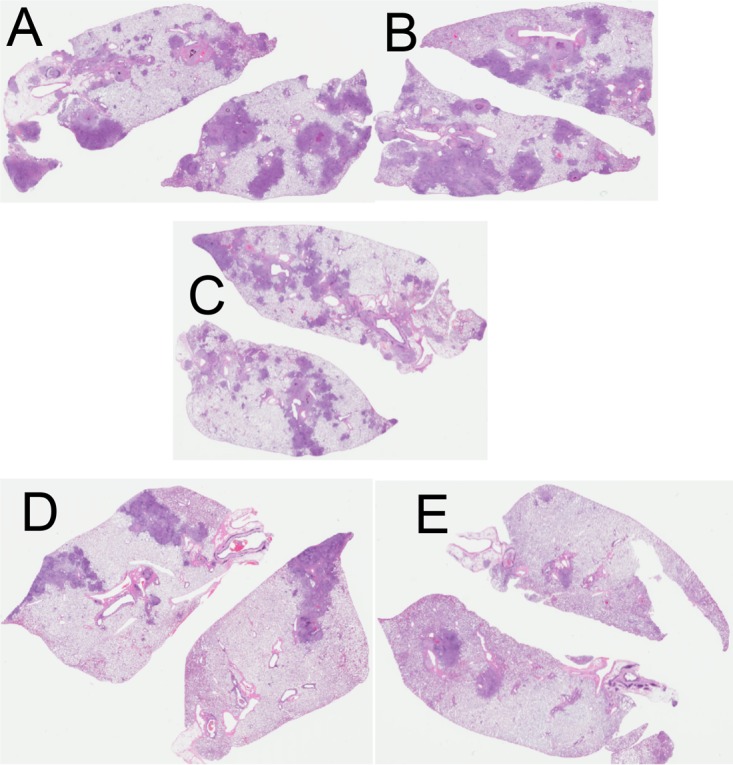
Whole-lung scans of representative samples stained with hematoxylin and eosin. (A) Saline control; (B) YVEC control; (C) 1-YU GI-19007; (D) animals vaccinated with BCG prior to challenge; (E) BCG-vaccinated animals given GI-19007 therapeutically. Scans shown are from animals infected 70 days earlier with 10 to 20 M. tuberculosis Beijing strain 212 bacteria.

Given the widespread use of the BCG vaccine across the world, we investigated if GI-19007 could facilitate protection if given therapeutically to BCG-vaccinated guinea pigs. Animals were given BCG and then 12 weeks later challenged with Beijing strain 212. Animals were vaccinated with 1 YU of GI-19007 as described above, and the bacterial load was determined on day 70 of the infection. As shown in Fig. 7, BCG protected strongly in the lungs, reducing the bacterial load by 1.4 log. No differences in lung bacterial load were seen in animals given both vaccines. However, while BCG limited bacterial dissemination to the spleen by 1.5 log, this was improved (*P* < 0.01) by another 1.1 log in animals given the two vaccines in combination. In terms of lung damage and pathology, as anticipated, BCG vaccination dampened the severity and numbers of lesions by itself (Fig. 8), although secondary lesion development remained evident, whereas while the overall lesion scores were comparable, far less secondary lesion development was seen in animals given both vaccines.

**FIG 9:**
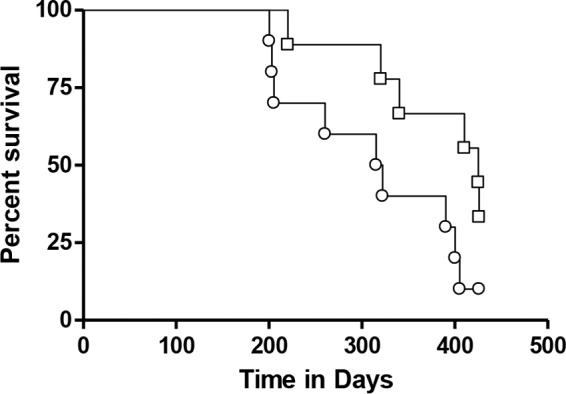
Kaplan-Meier survival curves for guinea pigs vaccinated with BCG prior to challenge (circles) compared to animals given BCG and then GI-19007 (squares) on days 10, 25, and 40 after challenge with Beijing strain 212. Through 300 days, survival in the double-vaccinated group was significantly improved compared to that of the BCG control group, but after this time the curves converged to the point this significance was lost. Each group contained 10 guinea pigs.

**FIG 10:**
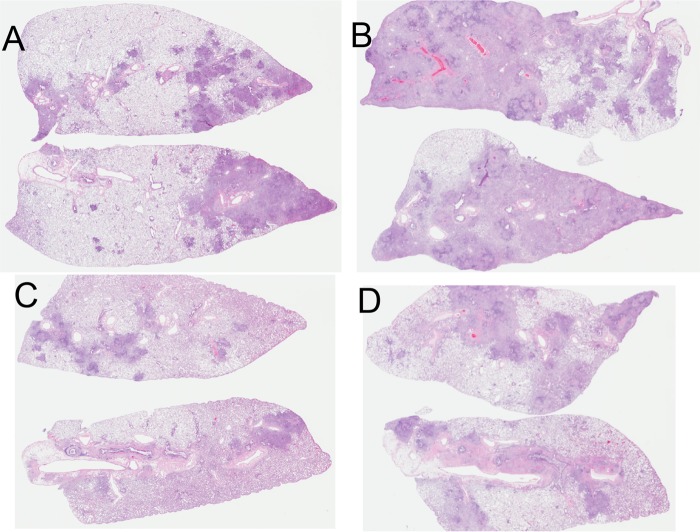
Whole-lung scans of representative samples stained with hematoxylin and eosin after harvest on day 330 of the infection. (A and B) BCG only; (C and D) BCG and then GI-19007 therapeutically.

Survival was monitored in a second group of animals in the same study (Fig. 9). If the study had been curtailed at day 300, we would have been able to report that BCG followed by GI-19007 given therapeutically significantly increased survival compared to that after BCG vaccination alone (histologic analysis on day 330 suggested this [Fig. 10]). However, at around the day 400 time point we observed increased mortality in both groups of animals, causing these curves to converge, which at this time we cannot explain (histologic examination suggested that lesions in the prime-boost group were less severe, but the sample size was too small to be conclusive).

## DISCUSSION

The results of this study show that a new candidate vaccine, GI-19007, delivered using a novel yeast-based platform and consisting of a fusion polypeptide made from four hypoxia-associated mycobacterial proteins (Rv1738, Rv2032, Rv3130, and Rv3841), was immunogenic in mice, generating both Th1 and Th17 responses. In the latter case, this included an expansion of CD8^+^ IL-17^+^ cells. Despite this, however, this formulation was ineffective as a prophylactic vaccine in the mouse model.

Because mice do not develop lung necrosis, we moved to the guinea pig model to evaluate any potential therapeutic activity, given our results with an earlier formulation based on these four proteins (7). Encouragingly, in the present study we observed reduction in the bacterial load in guinea pigs infected with the virulent Western Cape Beijing strain 212, and this was associated with a reduction in lung pathology, including the development of secondary lesions, a prominent feature seen in unvaccinated controls. As with our previous study, this was also associated with improved survival in about one-third of these animals. Finally, given the widespread usage/coverage of BCG, we also examined the effect of therapeutic vaccination in infected guinea pigs that had previously been vaccinated with BCG. This combination did not boost the effect on reducing the lung bacterial load, which was already substantial in the BCG controls (as also seen previously) by itself, but we did observe a reduction in secondary dissemination to the spleen, further emphasizing our view that this may be the central property of the GI-19007 vaccine. In terms of survival, guinea pigs given BCG began to die after day 180, but this event was substantially delayed in animals also given the therapeutic vaccination.

Despite this, this improvement in survival was steadily lost after day 400. The reason for this is unclear, but immunosenescence and/or the durability of the immune response could be contributing factors based on previously published studies in tumor and viral infection scenarios. In both the murine and guinea pig studies presented in this report, immunizations did not exceed three in number. In contrast, in tumor models it was reported (25) that each of four immunizations led to progressively increased cytotoxic T cell responses, and a further model (26) concluded that 10 immunizations were more effective than six.

Moreover, in humans, a whole-yeast-based immunotherapy directed to hepatitis C virus (HCV) combined with the then standard of care, type 1 interferon plus ribavirin, led to a significant 18% improvement in end-of-treatment (EOT) virologic responses compared to the standard of care alone (27). Of particular importance to the present discussion is that clinical trial subjects were treated monthly for 48 weeks total, and even after that extended treatment protocol some patients who had attained undetectable levels of virus at EOT relapsed 24 weeks later. Thus, both the frequency of immunizations and continuance of treatment are valid areas to be explored in future studies of TB, as they provide logical approaches that could improve efficacy.

Progress in the general status of therapeutic vaccines for tuberculosis remains modest, as comprehensively reviewed elsewhere (28). The majority of data we have so far have been obtained in the mouse, which comes with the caveat that this species does not develop the caseating necrosis seen in guinea pigs or humans (29). The first serious attempt to generate a therapeutic vaccine (initially aimed at latent tuberculosis) was made by Cardona and others, who developed the RUTI vaccine, which was culture stressed bacilli that then were fragmented, detoxified, and delivered in a liposomal formulation (30–32). This vaccine was shown to facilitate chemotherapy while promoting the expansion of CD8 cells (the latter observation was analogous to our observations here). Another candidate, *Mycobacterium w*, now renamed *M. indicus pranii*, was also shown to facilitate chemotherapy in BALB/c mice infected with H37Rv, but it was ineffective in mice infected with three multidrug-resistant strains (despite the authors' claims to the contrary) (33). In another study (34), the prophylactic vaccine H56 (Ag85B, ESAT-6, and Rv2660) reduced regrowth in CB6F1 mice after incomplete chemotherapy (our own reservations about this protocol and results are discussed in detail elsewhere [28]). Finally, the vaccine ID93, which has strong prophylactic activity, also has been shown to facilitate chemotherapy to a small degree (19), resulting in reduced lung pathology and improved survival, even in SWR mice in which untreated infection is progressive and fatal (35).

Our laboratory has previously reported two studies using the guinea pig model. In the first (36), we showed that a fusion protein (F36) comprising ESAT-6 and acylated Rv1411 (a potent TRL2 agonist) given 10 days after infection with H37Rv gave a 0.74-log reduction in the bacterial load at day 40. This was associated with some reduction in lung pathology scores but did not translate into improved survival. More recently, as noted above, our four hypoxia-associated proteins given in a GLA adjuvant formulation did not alter the lung bacterial load in guinea pigs infected with high-virulence clinical isolates but appeared to prevent secondary lesion development and improved survival in a percentage of the vaccinated animals (7).

Much of the data analyzing the protective effect of new prophylactic vaccines is based on short-term assays in which the lung bacterial load is reduced 1 to 2 logs and on survival assays in which, when the widely used laboratory strains are the challenge, 100% survival sometimes can be demonstrated. On the other hand, our own studies have shown (37) that even when very effective short-term protection can be observed when animals are infected with relevant Beijing or Haarlem strains, eventually these animals will die from the infection (an observation that obviously questions the use of laboratory strains in screening new vaccines [5, 28, 38, 39]). In the case of using vaccines therapeutically the bar is set even higher, since the infection has time to become established and, in the case of guinea pigs (and presumably humans), initiate mechanisms that drive necrosis in infected lesions. Indeed, only a single study has ever claimed that a therapeutic vaccine can cure the animal, and it may be that the best we can hope for is a candidate that slows the course of the infection, and reduces lung damage to some extent, while a parallel search is made for an effective chemotherapeutic regimen.

GI-19007 seems to have this property, and it was encouraging to see a reduction in lung bacterial counts, something we did not observe previously in studies using GLA as the vaccine delivery vehicle. In this regard, we should note that while our earlier studies concluded that once lung lesion necrosis is established it may be very hard to reverse, this may not always be the case. In our previous study in which the four hypoxia-associated proteins were given in the GLA adjuvant platform, about 30% of these animals survived past 200 days, and much of the lung necrosis had been replaced by fibrosis. In the current study, a similar increase in survival in a comparable fraction was observed. The basis for why these long-term survival effects are partial is unknown, but it may reflect the outbred nature of the animals used.

An obvious caveat to our results was the actual platform dosage, and our data clearly showed that higher doses (3 to 10 YU) of the vaccine were actually detrimental. This included lung histopathology in which lesions were very large and lymphocytic, a possible consequence of the known Th17-inducing ability of the Tarmogen platform. While it is not yet completely clear how important the Th17 component is in tuberculosis vaccine design, a large and sustained response is likely to contribute to cell influx and lung consolidation, which can be detrimental. If so, this suggests that the balance between Th1 and Th17 induction by vaccines is critical. One explanation for our data is that the delivery platform drives the production of IFN-γ effectively, but low doses are less effective at driving the Th17 pathway. Of course, in this regard, Th17 induction is one of several elements of vaccine design in tuberculosis that is not yet being adequately addressed.

An unexpected observation in our mouse vaccination studies was the induction of a substantial subset of CD8 T cells staining positive for IL-17. These have occasionally been observed in various fungal and protozoan disease states (40–42) and were found in one study within tuberculosis-induced pleural infusions (43). As yet, it is unclear whether their presence is beneficial. In terms of host immunity to tuberculosis, the field has focused to date on protective CD8 cells, but while CD8 cells can prolong survival (44), they only represent ∼10% of the lung response in infected mice (45), and mice lacking these cells show no differences in disease outcome until well into the chronic phase of the disease (46). In addition, a recent attempt to skew the response to vaccines to a dominant CD8 response had no obvious benefit (47). Our results indicate that under the correct conditions CD8 cells can be a source of IL-17 and thus may be helping to direct the correct cellular influx into lesions rather than simply being an IFN-γ-secreting protective T cell.

A consistent observation throughout our series of studies using our therapeutic vaccine candidates was the reduction, and in some case complete prevention, of the development of secondary lesions. After a site of infection is established (in our working model [48] in the interstitium rather than the alveolus), a primary lesion begins to develop. Small pockets of necrosis begin to appear soon afterwards as neutrophils entering the lesions from the blood degranulate, and this may also herald the beginnings of dissemination, as it is apparent in this animal model that some bacilli reach the adjacent draining lymph nodes soon afterwards, resulting in rapid lymphadenopathy (49). Others probably drain into lung lymphatic vessels, which follow a downward pattern toward the lung pleura, resulting in the establishment of secondary lesions predominantly in that region as well as cellular responses in the vessels themselves (lymphangitis) (50). By now the primary immune response is in full operation, so the secondary lesions do not become necrotic but instead fill with lymphocytes and macrophages, becoming very large and consolidating.

GI-19007 had no prophylactic ability in our studies, indicating that the four proteins were not among the targets of the initial emerging immune response, which is hardly surprising since the bacilli at this stage are proliferating freely and have yet to be exposed to stressful antimicrobial conditions. This also is consistent with our observations that the therapeutic vaccination had no effect on the developing primary necrosis, suggesting that GI-19007 is inducing T cells, probably a mixture of Th1 and Th17 cells based on our results, that are beginning to recognize stress proteins generated by bacilli that now find themselves in this developing necrotic environment. If Th17 cells are key to this (and unfortunately as yet there is no way to directly test this hypothesis in guinea pigs other than looking at gene expression [51]), then their potential mechanism of action could be the recruitment of more cells to the perimeter of primary granulomas (“plugging the gaps”) or into the draining lymphatics, resulting in the outcome that bacterial dissemination from these necrotizing structures is prevented, as the immunopathologic evidence strongly suggests. In addition, if this hypothesis is correct, it would diminish hematogenous dissemination to organs such as the spleen, as our result described above suggests.

Hence, if the primary activity of GI-19007 is prevention of dissemination of bacteria from lesions, then this activity may also be of benefit in the prevention of relapse occurring in chemotherapy-treated animals in which treatment was not completely sterilizing, an event seen in the guinea pig given even the most potent drug combinations (52). This potential, in addition to direct facilitation of chemotherapy by GI-19007, has yet to be explored.

## MATERIALS AND METHODS

### Animals.

Specific-pathogen-free female C57BL/6 mice, 6 to 8 weeks old, were purchased from the Jackson Laboratories (Bar Harbor, ME). Mice were maintained in the biosafety level III facilities at Colorado State University and were given sterile water, chow, bedding, and enrichment for the duration of the experiments. Specific-pathogen-free, female outbred Hartley guinea pigs (450 to 500 g in weight) were purchased from the Charles River Laboratories (North Wilmington, MA) and held under barrier conditions in a biosafety level III animal laboratory. Prior to inclusion in these studies, animals were appropriately acclimated and then microchipped for individual animal identification. The specific-pathogen-free nature of the mouse and guinea pig colonies was demonstrated by testing sentinel animals. All experimental protocols were approved by the Animal Care and Use Committee of Colorado State University. Uninfected female C57BL/6 mice were also used for immunogenicity studies in a biosafety level II vivarium under protocols approved by GlobeImmune's IACUC and in compliance with assurance A4700-01, issued to GlobeImmune by the National Institutes of Health Office of Laboratory Animal Welfare.

### Infections.

The laboratory strain H37Rv and the virulent Western Cape (24) clinical strain 212 of M. tuberculosis were used in these studies. The strains were grown in 7H9 broth containing 0.05% Tween 80, oleic acid-albumin-dextrose-catalase (OADC), and glycerol. When cultures reached an optical density at 600 nm of 0.600 to 1.00 they were bottled and frozen, and the viable bacterial concentration was determined by plating. Mice were infected using a Glas-Col aerosol generator (Glas-Col, Terre Haute, IN), calibrated to deliver 50 to 100 bacteria into the lungs. A Madison chamber aerosol generation device was used to expose guinea pigs, calibrated to deliver 10 to 20 bacilli into the lungs. Bacterial loads in target organs were determined by plating serial dilutions of individual whole-organ homogenates on nutrient 7H11 agar. CFU were counted after incubation for 3 weeks at 37°C in humidified air.

### Construction of the GI-19007 vaccine.

Recombinant S. cerevisiae cells expressing the four hypoxia-associated proteins (7) were engineered by methods similar to those previously described (21). The fusion gene in GI-19007 was produced by subcloning a synthetic DNA fragment encoding an in-frame fusion of Rv1738, Rv2032, Rv3130, and Rv3841 into a 2μm yeast-Escherichia coli shuttle vector (pGI-100) (22). The fusion gene was placed under the control of the copper-inducible *CUP1* promoter (Fig. 1). The N-terminal sequence MADEAP was added to enhance metabolic stability, and a C-terminal hexahistidine tag was appended for facile detection by Western blotting and enzyme-linked immunosorbent assay (ELISA). The cloned DNA insert was sequenced and the plasmid was transfected into S. cerevisiae yeast (W303α) (21). Transfectants were selected on solid complete medium lacking uracil (Teknova, Hollister, CA). Colonies selected from synthetic complete agar plates lacking uracil (UDA) were used to inoculate liquid medium containing 20 g/liter glucose, 6.7 g/liter yeast nitrogen base, and 2 mg/ml each of adenine, histidine, tryptophan, and leucine (U2). Liquid yeast cultures were grown to mid-exponential phase (2 YU/ml) and induced with 500 μM CuSO_4_ for 3 h. Yeast were harvested by centrifugation for 10 min at 5,700 × *g*, washed once in phosphate-buffered saline (PBS), and heat inactivated at 56°C for 1 h. The yeast cells were washed thrice in PBS and resuspended in PBS at 20 YU/ml (1 YU = 10^7^ Tarmogen yeast cells). W303α parental control yeast were transfected with empty plasmid vector pGI-100 to create a control yeast (YVEC) for use as a negative-control immunogen. Total yeast protein was extracted using a glass bead rupture method, and the expression of 4-hypoxia fusion protein was quantified (22) by Western blotting using an anti-(His)_6_ tag monoclonal antibody (Novagen, Madison WI).

### Vaccinations.

Mice were vaccinated with BCG Pasteur at a dose of 1 × 10^6^ bacilli by the subcutaneous route. Guinea pigs were vaccinated with 1 × 10^4^ bacilli by the intradermal route or with 1 to 3 YU of GI-19007 by the intradermal route.

### Flow cytometry.

Mice were euthanized by CO_2_ asphyxiation, and the thoracic cavity was opened. The lung was cleared of blood by perfusion through the pulmonary artery with 10 ml of ice-cold PBS containing 50 U/ml of heparin (Sigma, St. Louis, MO). Lungs were aseptically removed, teased apart, and treated with a solution of DNase IV (30 μg/ml; Sigma Chemical) and collagenase XI (0.7 mg/ml; Sigma Chemical) for 30 min at 37°C. Erythrocytes were lysed with Gey's solution (0.15 MNH_4_Cl, 10 mM HCO_3_), and the cells were washed with Dulbecco's modified Eagle's minimal essential medium. Total cell numbers were determined by flow cytometry using BD liquid counting beads, as described by the manufacturer (BD Pharmingen, San Jose, CA). Single-cell suspensions of lung from each mouse were resuspended in PBS (Sigma-Aldrich) containing 0.1% of sodium azide and 4% bovine serum albumin (BSA). Fc receptors were blocked with purified anti-mouse CD16/32. Cells were incubated in the dark for 25 min at 4°C with predetermined optimal titrations of specific antibodies. Antibodies were purchased from BD Pharmingen. Samples were analyzed on a Becton Dickinson LSR-II instrument, and data were analyzed using FACSDiva v7.0 software. Individual cell populations were identified according to the presence of specific fluorescence-labeled antibodies. All of the analyses were performed with acquisition of a minimum of 300,000 events. To detect IFN-γ-positive or IL-17-positive lymphocytes elicited by TB and/or yeast antigens, cells were initially stimulated for 4 h at 37°C with 1× cell stimulation cocktail (eBioscience) diluted in complete Dulbecco's modified Eagle's medium. Thereafter, cells were stained for cell surface markers as indicated above and then fixed and permeabilized using a Fix/Perm and Perm wash kit (eBioscience). Thereafter, cells were incubated for 30 min at 4°C with FcBlock plus anti-IFN-γ (clone XMG1.2; eBioscience), rat anti-mouse IL-17 (clone TC11-18H10), or the respective isotype control.

### Immunogenicity and epitope mapping for TB antigen-specific T cell responses.

In various studies, 1 to 5 YU of GI-19007 in 50 μl of PBS was administered intradermally to 5- to 7-week-old female C57BL/6 mice (5 to 7 mice per treatment group). Immunization was performed at 2 sites (2.5 YU in each flank) on days 0 and 56. Ten days after the second immunization, the mice were euthanized by CO_2_ asphyxiation and splenocytes from pooled spleens from all mice in each group and prepared as cell suspensions with ammonium chloride-potassium (ACK) lysis as previously described (20). A total of 200,000 cells were incubated in a 200-μl volume of complete RPMI medium with or without the 4 hypoxia antigens in round-bottom 96-well tissue culture plates; triplicate wells were processed in most assays. Incubation with antigens was at 37°C in a humidified 5% CO_2_ chamber for 4 days, followed by transfer of cell-antigen mixtures to 96-well murine IFN-γ ELISpot plates for 24 h (23). ELISpot assay plates were developed according to the plate manufacturer's instructions, and spot enumeration was conducted by Cellular Technologies, Ltd. (Shaker Heights, OH). For epitope mapping studies, the procedure described above was followed except that immunization with GI-19007 was on days 0 and 7, with *in vitro* stimulation of lymph node cells with 1 to 20 μM peptide occurring on day 17. All peptides were synthesized by Peptide 2.0, Inc., at 98% purity and resuspended in dimethyl sulfoxide (DMSO) at 10 to 40 mg/ml. Final DMSO concentrations in working assays were below 0.1% in all experiments.

### Pathology analysis.

The lung lobes from each guinea pig were fixed with 4% paraformaldehyde in PBS. Sections from these tissues then were stained using hematoxylin and eosin. Scanned sections then were reviewed for the presence of primary and secondary lesions. Primary lesions were identified based on distribution (more likely to be around blood vessels or larger airways or in central tissues) and showing evidence of developing necrosis. These lesions often contain various numbers of lymphocytes, macrophages, and neutrophils. Secondary lesions were more likely to be close to the lung pleura, were often much larger, and consisted of large numbers of lymphocytes, with some macrophage fields but no obvious necrosis.

### Statistics.

Differences in bacterial loads and host responses were determined using analysis of variance statistics. The ability of the vaccines to improve long-term protection was determined in Kaplan-Meier survival studies. Both analyses were performed using Prism software v4 (GraphPad, San Diego, CA). Study endpoints were determined by periodic weighing and visual observations that were based on a modified Karnofsky scale. Using this, a guinea pig was euthanized if the animal showed extensive labored breathing, was lethargic, had a matted or scruffy coat, had darkened eye color, was nonresponsive, and/or if the weight loss was greater than 20% of the weight of the animal recorded at the time of challenge.

### Data availability.

Materials relevant to this article will be made available in a timely fashion, at reasonable cost and in limited quantities as feasible, to members of the scientific community for noncommercial purposes under a material transfer agreement.

## Supplementary Material

Supplemental material
