# Supplementary material for: GI-19007, a Novel Saccharomyces cerevisiae-Based Therapeutic Vaccine against Tuberculosis
Source: Clin Vaccine Immunol. 2017 Dec 5;24(12):e00245-17. doi: 10.1128/CVI.00245-17 (PMC5717186; doi:10.1128/CVI.00245-17)
Supplement: Supplemental material [file supp_24_12_e00245-17__index.html]

Supplemental material 

# GI-19007, a Novel Saccharomyces cerevisiae-Based Therapeutic Vaccine against Tuberculosis

## Supplemental material

- Supplemental file 1 -

  Fig. S1. Demonstration of proof of principle that the Tarmogen platform can be used to deliver mycobacterial antigens. Fig. S2. Identification of epitopes in the constructed fusion vaccine.

  PDF, 133K
