# Supplementary material for: GI-19007, a Novel Saccharomyces cerevisiae-Based Therapeutic Vaccine against Tuberculosis
Source: Clin Vaccine Immunol. 2017 Dec 5;24(12):e00245-17. doi: 10.1128/CVI.00245-17 (PMC5717186; doi:10.1128/CVI.00245-17)
Supplement: Supplemental material [file CVI.00245-17_zcd012175552s1.pdf]

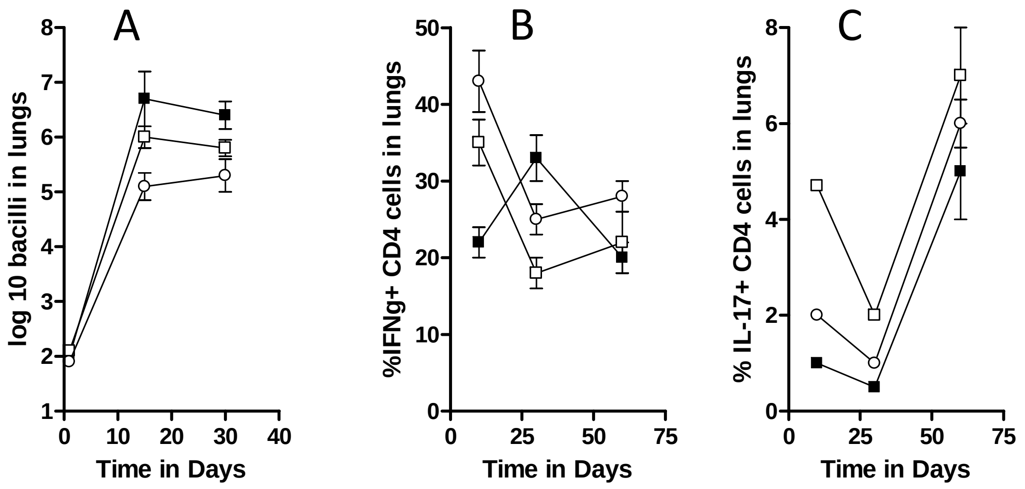

Supplementary Figure 1. Demonstration of proof of principle that the Tarmogen platform can be used to deliver mycobacterial antigens. Mice were vaccinated with Ag85A delivered in the yeast platform (open squares) and compared to BCG vaccinated mice (circles) or saline controls (closed squares) after low dose aerosol challenge with strain H37Rv. [A] bacterial load in lungs, [B] Percentage of CD4 cells staining positive for gamma interferon, [C] Percentage of cells staining as TH17 cells. Empty yeast (YVEC) had no protective effect by itself.

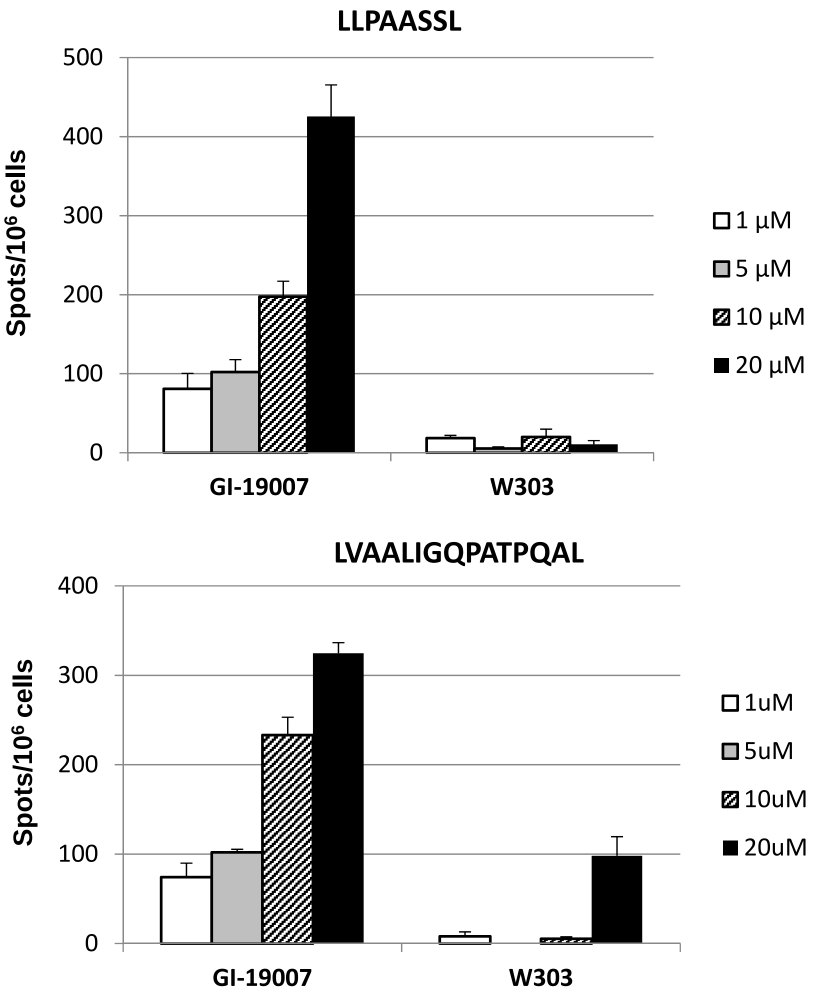

Supplementary Figure 2. Identification of epitopes in the constructed fusion vaccine. C57BL/6 mice were immunized intradermally with 1 YU of GI-19007 or parental yeast W303α. Pooled inguinal lymph node cells were stimulated in vitro with libraries of 9-mer and 15-mer peptides or medium alone and transferred to murine IFNg ELISpot plates for 24 hours. Peptide LLPAASSSL is an 9-mer Class I peptide located resides in Rv3130c (aa213-220), and peptide LVAACLGQPATPQAL is a 15-mer Class II peptide located in Rv2032 (aa284-298). The magnitude of IFNg response was dependent on the dose of peptide used for stimulation. Bars: open, 1 µM peptide; gray, 5 µM; diagonally shaded, 10 µM; and solid black, 20 µM. The average of quadruplicate wells is shown [±SEM].
